# Reports from the Records of the Atlanta Medical Society

**Published:** 1856-05

**Authors:** E. Hillyer


### ARTICLE V.

*Reports from the Records of the Atlanta Medical Society.* By E. Hillyer, M. D., Secretary. April 3d, 1856.

*Placenta Prævia.*—The regular subject of discussion, Placenta Prævia, was taken up in order.

Dr. Boring said that when the Placenta was placed over the os uteri, he preferred the course recommended by Ramsbotham, viz.: when the os was dilated, or dilatable, to partially detach the placenta, and carry the hand up between the membranes and the side of the womb, then perforate the membranes, seize the feet, and deliver by turning. He had recently seen a plan of procedure mentioned in the Virginia Medical and Surgical Journal, in which it was advised to place the woman in the erect posture, so as for the head of the child to press upon the cervix uteri, and thus arrest the hemorrhage by mechanical means. Dr. Boring was disposed to think that there was much plausibility in this idea.

Dr. Darnell recollected to have had but three cases to occur in his practice. In two of the cases, the placenta was placed only partially over the os uteri. Both the patients were much exhausted from the loss of blood when he saw them. He delivered them by partially detaching the placenta, and then turning. In one case the child was born dead; in the other, the infant lived, but was quite feeble at birth; in the third case, he found the placenta quite centrally placed over the os uteri. The hemorrhage was very violent and alarming. He immediately gave a large dose of ergot; and upon the authority of Dr. Simpson of Edinburgh, he entirely detached the placenta from the uterine surface, with the view of the immediate arrestation of the hemorrhage. He stated that he was much gratified to find that the hemorrhage was greatly diminished as soon as this was done. His fingers were constantly kept within the cervix to promote uterine contraction, and to aid in stopping the flow by compression. He had generally found that when the flow was considerable, the os made but little resistance.

In two hours after the separation, the placenta came away, soon followed by the child, which was dead.

The Secretary remarked that he would be very unwilling to resort to this means of arresting the hemorrhage, for in such a case the child must inevitably be sacrificed.

Dr. Darnell said he did it because the danger of the mother was imminent, and the life of the child had to be sacrificed for the mother, an[illegible] this was the most certain means he knew of for arresting the hemorrhage; he did not think the tampon would have done it; but this means was never to be resorted to unless the danger of the mother was so great as to demand the death of the child.

Dr. Logan remarked that he had no practical knowledge of this subject, it not having occurred to him to treat a case of placenta prævia, but that he had laid out for himself a plan of proceeding—in the event of meeting with such a difficulty—in the adoption of the propriety of the course recommended by Mr. Braithwaite, which he says that he has pursued for twenty years, in a very considerable number of cases, with not more than two fatal results. Dr. L. then proceeded to state Mr. Braithwaite's views, which are as follows: If there is not a sufficient dilation of the os uteri to permit the operation of *turning*—which all admit to be the proper mode of proceeding, where it can be accomplished—he at once introduces a tampon of soft linen, lint, or other suitable material, accurately adjusting it to the parts, so as to form an effectual plug; the vagina being filled up, there can be no escape of blood through it, and hence it must accumulate immediately around the bleeding vessels, its entrance into the uterine cavity being effectually prevented by the placenta and other contents.

The advantages (stated by Mr. Braithwaite) of this plan, were the promotion of uterine action, ensuring the safe dilation of the os, preserving the strength of the mother by the arrest of the threatening hemorrhage, and obviating the necessity of endangering the life of the child, most certainly involved in the plan of Professor Simpson.

He also remarked that Mr. B. explained the use of the plug upon what he termed the hydraulic principle; the blood being prevented from passing through the vagina by the presence of the tampon, and as already stated, not being allowed to enter the uterus, as soon as the blood had reached the orifices of the bleeding vessels, a coagulum would be formed, and the flooding arrested.

He also mentioned incidentally, that Mr. Braithwaite considered this plan equally applicable to the treatment of uterine hemorrhage, after delivery, where *the bleeding vessels were situated at the cervix*, in which he was disposed to concur—fully aware, however, of the insuperable objections to the use of the tampon after delivery under other circumstances—but let this be as it might, he believed it an *impossibility* that there could be any serious accumulation of blood within the uterine cavity, in cases of placenta prævia, *before delivery*, and where the membranes were unruptured, taking into consideration the obstacles presented; by the occupied uterus, the hydraulic principle (suggested by Mr. Braithwaite) and the coagulability of blood.

Dr. Coe had only one case to occur in his practice, which was only a partial attachment over the os uteri. He was in favor of using the tampon in cases where the os was undilatable, and as long as the hemorrhage could be controled by it, he was certain that the entire separation of the placenta would arrest the hemorrhage, but it was decidedly objectionable on the child's account, unless delivery should follow immediately after the detachment of the placenta.

Dr. Boring further said he was opposed to the use of the tampon; he did not think it safe, and that it could not be relied on.

Dr. J. G. Westmoreland said the first thing to be determined, was the possibility of saving both the mother and child. If the hemorrhage was not violent and threatening, he was in favor of application of the tampon, and give the child a chance of life; but on the other hand, if the hemorrhage was very violent, so as to cause the immediate prostration of the mother, and there was no hope of saving her but by the detachment of the placenta, he did not think the life of the child should be regarded. In case the delivery had been accomplished, and the hemorrhage continued, he was decidedly averse to the tampon, as the womb would contain such an amount of blood as the patient could not bear the loss of, even should the hemorrhage be from the cervix; it would fill up that organ.

Dr. Logan further stated that he concurred with Mr. Braithwait, that where the bleeding surface was located in the cervix, that it was not probable that the womb would fill with blood.

[It is a generally conceded opinion, that where the os uteri is dilated or dilatable, the best mode to overcome this difficulty arising from the mal position of the placenta, is that of the partial separation and delivery by turning.

But suppose we have a case where the os is rigid and unyielding, and our judgment is against forcing it. Here we have the course recommended by Simpson, and followed by Dr. Darnell, viz: the entire separation of the placenta upon the supposition that the hemorrhage will cease. Some gentlemen think that they can control the flow of blood with more certainty by this course than by the use of the tampon; and while they admit they would use the tampon in a less degree of hemorrhage, from the placenta being placed *prævia*, they separate entirely upon the ground that the blood will be more effectually stopped than by the tampon.

It is a well known fact, that when the placenta is detached from the uterus, and it does not immediately contract, there will be a very profuse and perhaps fatal hemorrhage. Should it be entirely detached from the fundus or body, there is some probability of checking the flow, because the uterus can contract upon the membranes or fœtus in case the waters are discharged, and such an amount of pressure will be produced upon the bleeding surface, as will effect the desired object.

Now, when it has been detached from over the os tincæ, instead of contraction, we have the exposed surface remaining without any condensation of its tissue, and has thus to remain or become more relaxed by the further dilatation of the os until the delivery is accomplished, and there must be a considerable degree of hemorrhage while this portion of the womb remains uncontracted. It is my opinion that the tampon is the most effectual means we have for this purpose. We can certainly so plug up the vagina that there will be no external hemorrhage.

Then is it probable that there will be any great degree of internal hemorrhage? I think not. The os uteri is undilated: otherwise, we should proceed to turn and deliver immediately. The waters not discharged, as in nine cases out of ten they are not, the vagina filled up with the tampon, the womb occupied entirely by the fœtus and the liquor amnii. Where there is the space sufficient to contain such an amount of blood as will endanger the life of the mother, if the placenta is entirely detached, the hemorrhage must continue from the surface of its attachment upon the womb. Even should the flooding be excessive and dangerous, the os being undilated, I should not hesitate to apply the tampon; because I think it much the most certain means of checking the hemorrhage, and then, perhaps, if skillful in turning, we save the life of the child. If we entirely detach the placenta, unless delivery immediately follow, the child must inevitably perish. Even admitting that the two modes of controling the hemorrhage are equal, is not the application of the tampon the best, for we do not add to the danger of the child by its use, while in the other we certainly kill it? While the tampon is in the vagina, we have time to reflect and determine calmly upon our future course. The os is in the mean time becoming more relaxed, so that when we shall remove the tampon some hours afterwards, we can proceed to partially detach the placenta, and turn and deliver as before described.—*Secretary.*]

